# Development of a Novel Virus-Like Particle-Based Vaccine Against PRV-1 Suitable for DIVA Strategies

**DOI:** 10.3390/v17121578

**Published:** 2025-12-02

**Authors:** Claudia Galleguillos-Becerra, Matias Cardenas, Yesseny Vásquez-Martínez, Francisca Tapia, Zulema Yañez, Tomas Cancino, Iván Valdés, Marcelo Cortez-San Martín

**Affiliations:** 1 Facultad de Química y Biología, Universidad de Santiago de Chile, Santiago 9170022, Chile; claudia.galleguillos@usach.cl (C.G.-B.); matias.cardenas.p@usach.cl (M.C.); yesseny.vasquez@usach.cl (Y.V.-M.); francisca.tapia@usach.cl (F.T.); zulema.yanez@usach.cl (Z.Y.); 2 Escuela de Medicina, Facultad de Ciencias Médicas, Universidad de Santiago de Chile, Santiago 9170201, Chile; 3 Laboratorios Veterquimica SA, Santiago 9200000, Chile; tcancino@veterquimica.cl (T.C.); ivaldes@veterquimica.cl (I.V.)

**Keywords:** *Piscine orthoreovirus*, VLP vaccine, cmyc epitope

## Abstract

*Piscine orthoreovirus* genotype 1 (PRV-1) is an emerging viral pathogen in salmon aquaculture that causes Heart and Skeletal Muscle Inflammation (HSMI), with high prevalence in salmon-producing countries such as Chile. A significant obstacle in PRV-1 vaccine development is the inability to culture the virus in vitro, which limits the scalability and production of traditional inactivated or DNA-based vaccine strategies. This study describes the development of a novel virus-like particle (VLP)-based vaccine against PRV-1. Recombinant VLP were produced by co-expressing the six structural proteins of PRV-1 (λ1, λ2, μ1, σ1, σ2, σ3) using a baculovirus-based expression system in insect cells. In addition, to enable differentiating infected from vaccinated animals (DIVA) strategies, the σ1 protein was modified by adding of a cmyc epitope tag. The results demonstrated that the native VLP vaccine (VLP6n) significantly reduced viral loads in Atlantic salmon challenged with PRV-1. Moreover, in rainbow trout, the cmyc-tagged VLP-like vaccine (VLP6c) elicited a specific antibody response against the cmyc epitope, allowing differentiation between vaccinated and naturally infected fish. Overall, this VLP-based vaccine platform represents a promising strategy for controlling PRV-1 prevalence in salmon-producing counties, supporting the implementation of serological surveillance programs.

## 1. Introduction

*Piscine orthoreovirus* (PRV), now called *Orthoreovirus piscis*, is a reovirus member of the *Orthoreovirus* genus and the etiological agent of Heart and Skeletal Muscle Inflammation (HSMI), which has been reported in several salmon-producing countries, including Chile, Norway, and Canada, where PRV is prevalent. However, clinical HSMI cases are less frequent [1]. Like all orthoreoviruses, PRV is a non-enveloped virus with a double capsid and an icosahedral structure, ranging from 60–80 nm in diameter [2]. It has a 10-segment double-stranded RNA genome, which is classified based on its electrophoretic mobility: three large segments (L1, L2, and L3), each 3.9 kb in length; three medium segments (M1, M2, and M3) ranging from 2.1 to 2.4 kb; and four small segments (S1, S2, S3, and S4) ranging from 1.0 to 1.4 kb, giving a total genome size of 23.32 kb [3,4]. Based on phylogenetic analysis using the S1 and M2 segments, PRV isolates can be classified into three different genotypes: PRV-1, PRV-2, and PRV-3, with PRV-1 strongly associated with HSMI [5]. Although the exact role of the PRV proteins remains unknown, homology studies with some orthoreoviruses, including mammalian (MRV) and avian reoviruses (ARV), have suggested that the λ1, λ3, µ2, σ2, λ2, µ1, σ3, and σ1 proteins might be the structural components of the viral particle [6]. The first four proteins (λ1, λ3, µ2, and σ2) are believed to form the inner capsid, while the remaining proteins might be part of the outer capsid [3].

To date, no suitable in vitro system exists for culturing PRV-1, which remains unculturable [7]. This limitation has strongly affected the development of specific treatments for HSMI and vaccines targeting PRV-1. However, an inactivated vaccine produced in vivo has been reported [8]. Nevertheless, this technique is time-consuming, expensive, and carries the risk of infection and virus replication resulting from the inactivation procedure [9]. Additionally, vaccination with a DNA vaccine encoding the salmonid Alphavirus replicon and the structural and non-structural PRV-1 proteins has been tested but failed to provide protection [10]. The lack of protection and the intrinsic risks associated with currently reported vaccine strategies highlight the need for novel vaccine platforms against PRV, such as recombinant antigens comprising immunogenic viral proteins. In this context, it has been observed that combining multiple antigens in a vaccine formulation, a synergy may exist between them. This effect is believed to enhance antigen presentation, resulting in better activation of both B and T lymphocytes [11], coupled with a stronger antibody production. This may occur because the antigens included in the formulation correspond to structural proteins of the target virus, allowing them to self-assemble into multiprotein complexes resembling the wild-type viral particle (virus-like particles, VLP) [12].

In the context of aquaculture, several VLP-based vaccines have been developed and tested against various fish pathogens. For instance, in 2021, a study showed that production of the grass carp aquareovirus structural recombinant proteins VP3, VP4, and VP8 spontaneously self-assembled into VLP with a morphology resembling the viral particle. Immunized carp also exhibited enhanced specific IgM production targeting the virus, which might have led to a higher survival rate (83.33%) after challenge [13]. Current data suggest that VLP elicit an effective immune response and serve as excellent candidates for vaccine development [14]. Additionally, VLP provide an attractive platform for displaying foreign epitopes or targeting molecules, and one or more immunogenic proteins are included, still resulting in the induction of specific antibodies [15]. Based on this principle, VLP vaccine candidates offer a promising strategy for differentiating infected from vaccinated animals (DIVA), and VLP can be constructed based on the need for serological surveillance. This is an interesting strategy for the most frequently detected viral pathogens, as is the case with PRV in Chile.

In terms of immunogenicity, studies have demonstrated that the PRV-1 σ1 and μ1 proteins can induce a humoral immune response when evaluating the production of specific antibodies in salmon [16,17]. In vivo challenge assays showed that the maximum production levels of Anti-PRV σ1 and μ1 were correlated with a reduction in HSMI lesions, which could indicate a protective effect. Although the structural proteins of PRV-1, including σ3 and λ2, can induce antibodies [16], there is currently no experimental evidence evaluating their protective role against PRV-1.

In this study, we developed a VLP-like assembly-based vaccine containing a mixture of six structural PRV-1 proteins—λ1, λ3, µ2, σ2, λ2, µ1, σ3, and σ1—produced in insect cells, which was used to immunize Atlantic salmon. The data showed that our vaccine could provide load viral protection, representing a novel approach for vaccine development against PRV-1 infection. Then, the σ1 protein was modified by adding cmyc epitope to VLP-cmyc production, thereby generating a specific humoral immune response in salmonids that allows for differentiation between vaccinated and non-vaccinated salmonids. The σ1 protein was selected for the insertion of the cmyc epitope because it is the surface-exposed fiber protein of PRV-1, responsible for host cell attachment and a primary target of neutralizing antibodies. Its external localization enables DIVA differentiation by anti-cmyc serology, whereas modifications to internal proteins could interfere with particle assembly. Similar strategies targeting surface fibers proteins have been successfully used in other orthoreovirus systems [6,18].

## 2. Materials and Methods

### 2.1. Virus and Cells

SF9 cells derived from *Spodoptera frugiperda* were cultured in Sf900II media (Gibco, Billings, MT, USA) 100 supplemented with 100 μg/mL penicillin/streptomycin (Gibco). High Five cells from *Trichoplusia ni* were maintained in Express Five media (Gibco) supplemented with 18 mM L-Glutamine and 100 μg/mL penicillin/streptomycin. Both cell lines were incubated in suspension or adherent culture at 28 °C and media was changed twice a week.

### 2.2. Generation of pFastBac Vectors and Recombinant Bacmids

pFastcBac-1 plasmids containing the λ1, λ2, μ1, σ2, σ3, and σ1 coding open reading frame (ORF) sequences from the Chilean PRV-1 isolate CGA280-05 (ID GenBank: KC795573.1) were synthesized by Genscript Co. ORF sequences were codon-optimized to insect cells, obtaining the pFastcBac_λ1, pFastcBac_λ2, pFastcBac_μ1, pFastcBac_σ2, pFastcBac_σ3, and pFastcBac_σ1. Recombinant bacmids were generated by transforming DH10Bac cells (Thermo Scientific, Vilnius, Lithuania) with each recombinant pFastBac plasmid, identifying positive colonies by blue-white screening using X-Gal-IPTG-supplemented agar plates. Subsequently, recombinant bacmids were purified by alkaline lysis. Briefly, recombinant clones were incubated overnight at 37 °C in Luria-Bertani (LB) media supplemented with 50 ng/µL kanamycin, 7 µg/µL gentamicin, and 10 µg/µL tetracycline. After incubation, bacteria were pelleted at 9000 rpm for 5 min, the supernatant was discarded, and the pellet was resuspended in resuspension buffer (15 mM Tris-HCl, pH 8, 10 mM EDTA, and 100 ng/mL RNase A). Then, cells were lysed with lysis buffer (0.2 M NaOH and 1% SDS) for 5 min at room temperature. After incubation, 300 µL of 1 M potassium acetate was added, and the samples were incubated for 30 min on ice. Finally, bacmids were precipitated by adding 1 mL of isopropanol followed by centrifugation at 14,000 rpm for 10 min at 4 °C. The pellet was washed twice with ethanol and resuspended in nuclease-free water. Insert transposition was then confirmed by PCR using M13 primers (M13 forward: 5′CCCAGTCACGACGTTGTAAAACG-3′; M13 reverse: 5′-AGCGGATAACAATTTCACACAGG-3′).

### 2.3. Cloning of Human Oncogene Cmyc Epitope in pFastBac_σ1 Vector

To obtain the pFastBac-σ1cmyc vector, the pFastBac-σ1 was used as a template, and cmyc was introduced using the primer S4_cmycFw (5’GGATCCATGCATAGATTTACCCAAGAAGACCATGTTA3′) and S4_cmycRv (5′GAATTCCTAGAGGTCTTCTTCGGAAATCAACTTCTGTTCGATGATGATC3′) primers. The PCR was performed using Phusion High Fidelity Taq Polymerase (NEB) according to the manufacturer’s instructions, yielding the σ1-cmyc insert. The σ1-cmyc PCR products and the pFastBac1 plasmid were digested with EcoRI (Thermo Scientific, Vilnius, Lithuania) and BamHI (Thermo Scientific, Vilnius, Lithuania), according to the manufacturer’s instructions, and the digested products were purified using the Wizard SV Gel and PCR Clean-Up System (Promega, Madison, WI, USA). Purified samples were ligated at a 3:1 insert/plasmid ratio. Subsequently, ligation products were transformed into DH5α cells using the methodology described by Van Die [18]. After the transformation a colony PCR was performed to the positive clone’s selection according to the previous methodology mentioned. Then, a restriction assay using EcoRI (Thermo Scientific) BamHI (Thermo Scientific) and sequencing (Macrogen, Seoul, South Korea) were performed to confirm correct insertion and reading frame of the cmyc epitope within the σ1 gene.

### 2.4. Recombinant Baculoviruses Rescue

To produce recombinant baculoviruses, SF9 cells were seeded at a density of 1 × 10^6^ cells/well in 6-well plates and transfected with each bacmid using the Cellfectin II reagent (Gibco). Briefly, cells were incubated at 27 °C for 1 h after seeding, and the media was then replaced with fresh Sf900II media. Then, 2 µg of recombinant bacmid were mixed with 100 µL of Sf900II media. On a different reaction, 6 µL of Cellfectin II was mixed with 100 µL of Sf900II. Subsequently, both mixtures were combined and incubated at room temperature for 30 min. After incubation, the transfection mixture was added dropwise onto the SF9 cells, and the plates were incubated for 4 h at 28 °C. Finally, the transfection media was discarded, and cells were supplemented with new SF900II media. Cells were maintained at 28 °C for 4 days or until 80% of cells exhibited cytopathic effect (CPE). Baculoviruses were aliquoted and expanded up to 3 times in SF9 cells as described in the Bac-to-Bac system manual (Thermo Scientific).

### 2.5. Detection of Recombinant Proteins by Western Blot

High Five in Express Five (Gibco) media supplemented cells were seeded on a 6-well plate at a density of 1 × 10^6^ and infected at a multiplicity of infection (MOI) of 1, then incubated at 27 °C. At 3 days post-infection (dpi), cells were pelleted for 5 min at 200 rpm, then resuspended in 100 µL of 0.1 mM phenylmethylsulfonyl fluoride (PMSF, Sigma, St. Louis, MO, USA). Cells were lysed by repeated freeze–thaw cycles, and total protein concentration was determined using the Bradford method (ThermoFisher, Rockford, IL, USA). Samples were normalized to 10 µg/line and analyzed by sodium dodecyl sulfate-polyacrylamide gel electrophoresis (SDS-PAGE) using 12% polyacrylamide gels. After electrophoresis, the proteins were electrotransferred to a polyvinylidene fluoride (PVDF) membrane, blocked with 5% non-fat milk in T-PBS buffer (0.1% Tween-20 in phosphate-buffered saline) overnight at 4 °C. Polyclonal antibodies against PRV-1 structural proteins (λ1, λ2, μ1, σ2, σ3, and σ1) were custom-produced by GenScript Co. (Piscataway, NJ, USA). The isolate used as a reference was CGA280-05. Each target sequence (λ1 NC_036477.1, λ2 NC_036468.1, μ1 NC_036470.1, σ2 NC_036473.1, σ3 NC_036472.1, and σ1 NC_036475.1) was optimized and synthesized in vitro and cloned into vector pET-30a(+) with His-tag. The recombinant protein expression was evaluated in transformant E. coli strain BL21(DE3) by SDS-PAGE and Western blotting. Each antibody was generated in rabbits immunized with the corresponding recombinant PRV-1 protein, expressed and purified by GenScript following their standard procedures, including antigen preparation, immunization, serum collection, and affinity purification. After blocking, membranes were incubated with one of the following custom-made antibodies: rabbit polyclonal anti-λ1 (1:10,000), anti-λ2 (1:10,000), anti-μ1 (1:1000), anti-σ2(1:1000), anti-σ3 (1:1000) or anti-σ1 (1:1000), all PRV antibodies was made in Genscript Co., and monoclonal anti-cmyc (1:1000) (Merck Cat. M4439) for 1 h at room temperature. Membranes were washed three times with phosphate saline buffer 0.01% p/p tween 20 T-PBS (Corning, Corning, NY, USA) (5 min each), followed by a 45 min incubation with 1:10.000 HRP-conjugated goat anti-rabbit secondary antibody (ThermoFisher, Rockford, IL, USA) at room temperature. Afterward, membranes were washed five times with T-PBS and developed using the Western Sun (Biorad, Pleasanton, CA, USA) chemiluminescence kit according to the manufacturer’s instructions.

### 2.6. Indirect Immunofluorescence Assay (IFA)

SF9 cells were seeded on polylysine-coated glass coverslips at a density of 4 × 10^5^ per well and infected with a passage 3 (P3) recombinant baculovirus. Non-infected SF9 cells and cells infected with a wild-type baculovirus were used as controls. At 48 hpi, the cell supernatant was discarded, and cells were washed three times with PBS (phosphate-buffered saline, Corning). Then, cells were fixed with 4% paraformaldehyde for 10 min at 4 °C, and slides were washed three times with PBS. After fixation, cells were permeabilized with 0.5% Triton X-100 in PBS for 10 min under gentle agitation. Coverslips were then washed three times with T-PBS, blocked with 3% bovine serum albumin BSA (Winkler) in T-PBS (blocking buffer), and incubated with a one of the following antibodies: rabbit anti-λ1 (1:1000), anti-λ2 (1:1000), anti-μ1 (1:1000), anti-σ2 (1:500), anti-σ3 (1:500), anti-σ1 (1:500), or monoclonal anti cmyc (1:1000) for 1 h at room temperature. After incubation, samples were washed three times with T-PBS and incubated with a goat anti-rabbit AlexaFluor 648-conjugated secondary antibody (ThermoFisher) in a dilution of 1:1000 in blocking buffer supplemented with 0.5 μg/mL 4′,6-diamidino-2-phenylindole (DAPI, Thermo Scientific) for 1 h at room temperature. Finally, slides were mounted on glass slides using 1,4-diazabicyclo[2,2,2]octane (DABCO) and imaged using a Zeiss LSM 800 confocal Microscope (Jena, Germany) at the Confocal Microscopy Unit of the Universidad de Santiago de Chile.

### 2.7. Co-Infection Assay and Simultaneous Protein Identification

High Five cells were seeded to 2 × 10^8^ cells in 100 mL of Express Five media supplemented with 18 mM L-glutamine (Sigma, St. Louis, MO, USA) and 100 µg/mL penicillin/streptomycin (Gibco). Subsequently, co-infections were performed with the recombinant baculoviruses, in order to obtain native-VLP (VLP6n), the following baculovirus inoculums were used: bac-λ1, bac-λ2, bac-μ1, bac-σ1, bac-σ2, and bac-σ3; in order to obtain cmyc-VLP (VLP6c), the following baculovirus inoculums were used: bac-λ1, bac-λ2, bac-μ1, bac-σ1cmyc, bac-σ2, and bac-σ3. A multiplicity of infection (MOI) of 1 for each baculovirus was used, except for bac-σ1 or bac-σ1c_myc, where an MOI of 2 was used. After 5 days post-infection (dpi), the cell suspension was centrifuged at 9000 rpm, and the cell pellet was resuspended in the media residual volume. Then, the recombinant proteins in the resuspended pellet were detected by Western blotting as previously described.

### 2.8. VLP Purification by Ultracentrifugation

After a 72 h co-infection assay to obtain native-VLP (VLP6n), the cell suspension was centrifuged for 10 min at 800× *g*. Cellular lysis was then performed using 1 mL of TNN buffer (50 mM Tris-HCl, pH 8.0, 150 mM NaCl, and 1% NP40), and the cellular debris was discarded by centrifugation at 500× *g* for 15 min. Then, 1 mL of the supernatant was added to a discontinuous gradient of 30–50% sucrose in PBS or CsCl gradient. The sucrose gradients were ultracentrifuged at 21,000 rpm for 2 h at 4 °C using an AH629 swinging-bucket rotor (Sorvall, Thermo Scientific). After ultracentrifugation, the interface between 30% and 50% was collected and stored on ice for further analysis. The purity of the sucrose gradient fraction was evaluated by SDS-PAGE followed by Coomassie Blue staining with no detectable major cellular contaminants. Western blot analysis using anti-σ1 antibodies further confirmed the identity of the viral protein present in the purified fraction. The CsCl gradient purification was performed according to Wessel et al. [8].

### 2.9. VLP Characterization by Transmission Electron Microscopy

To visualize the purified native-VLP, 5 μL of the interface suspension from the sucrose discontinuous gradient or the virus-containing fraction from the CsCl gradient was added to a 400-mesh nickel grid with a Formvar film (polyvinyl formaldehyde in 1,2-dichloroethane-chloroform) and left to rest for 1 min. After washing it three times with PBS, it was contrasted with 1% uranyl acetate for 1 min. Once dry, the sample was visualized using a transmission electron microscope (TEM). To visualize the co-infected cell cultures using electron microscopy, infected cells were embedded in acrylic resin. To this end, 1 mL of cell suspension co-infected with bac-λ1, bac-λ2, bac-μ1, bac-σ2, bac-σ3, bac-σ1, or bac-σ1c_myc baculoviruses was centrifuged at 500× *g* for 1 min. The supernatant was discarded, and cells were washed with PBS prepared in ultrapure water (Invitrogen, Carlsbad, CA, USA). Cells were then fixed for 2 h with 4% paraformaldehyde and 0.4% glutaraldehyde in PBS. After fixation, the supernatant was removed by repeated washes with PBS, and the cells were incubated overnight at 4 °C. Cells were then washed with Milli-Q water and embedded in 2% Low Melting Point (LMP) agarose. Samples were dehydrated by incubation for extended periods at increasing ethanol concentrations (up to 100%) at 4 °C. Subsequently, samples were resin-embedded by performing incubations in increasing resin concentrations, reaching 100% resin. Finally, three hours of incubation at room temperature with pure resin were performed, followed by polymerization at 55 °C for 24 h. Once the cells were embedded, ultra-thin sections of 60 nm were cut using an ultramicrotome (Leica Ultracut UCT, Wetzlar, Germany) and negatively stained using uranyl acetate. Samples were imaged using a Philips Tecnai Transmission Electron Microscope at the Advanced Microscopy Unit of the Pontificia Universidad Católica de Chile.

### 2.10. Antigen Quantification

For antigen quantification, the concentration of the σ1 protein in the co-infection cellular extract was determined by ELISA. A purified recombinant σ1-His protein (>90% purity), produced and purified by GenScript Co. (Piscataway, NJ, USA) via nickel affinity chromatography, was used as the standard for the ELISA. The σ1 recombinant protein (Genbank ID: NC_036475.1) was expressed in *E. coli* as a His-tagged. Known concentrations of this purified antigen (600 ng to 0.006 ng per well) were used to construct the standard curve. At 5 days post-infection (dpi), the co-infection cellular suspension was centrifuged at 3500 rpm, and the cell pellet was resuspended in the residual volume. Standard curve dilutions were prepared in PBS with decreasing concentrations of the recombinant σ1 oligopeptide: 600 ng, 60 ng, 6 ng, 0.6 ng, 0.06 ng, and 0.006 ng. Then, 100 μL dilution was used to coat microtiter plates overnight at 4 °C. Simultaneously, microtitration plates were incubated with 1:10, 1:100, and 1:1000 dilutions of the co-infection cellular extract overnight at 4 °C. After the incubation period, the plates were washed with PBS, and 100 μL of a 2% BSA blocking solution in PBS was added. Then, the plates were incubated with 1:1000 primary anti-σ1 rabbit antibody for 1 h at room temperature. After incubation, the plates were washed three times with PBS and incubated for 1 h at room temperature with a 1:10,000 HRP-conjugated anti-rabbit secondary antibody (ThermoFisher). Finally, 100 μL of 3,3′,5,5’-tetramethylbenzidine (TMB, Invitrogen) was added to each well for 15 min. The reaction was stopped by adding 100 μL of 1 M hydrochloric acid (Winkler). After calibration with the blank control, plate readings were performed by determining the OD value of the samples at 450 nm using the NanoQuant Infinite M2000 pro microtitration plate reader (TECAN, Grödig, Salzburg, Austria)

### 2.11. Vaccine Antigen Preparation

Vaccine antigens were prepared by seeding 2 × 10^8^ High Five cells in 100 mL of Express Five media supplemented with 18 mM L-glutamine (Sigma) and 100 µg/mL penicillin/streptomycin (Gibco). Subsequently, cells were co-infected with bac-λ1, bac-λ2, bac-μ1, bac-σ1 or bac-σ1c_myc, bac-σ2, and bac-σ3 baculoviruses at an MOI of 1 each, except for bac-σ1 or bac-σ1c_myc, in which an MOI of 2 was used instead. At 5 days post-infection (dpi), cell suspensions were centrifuged at 9000 rpm, the supernatant was discarded, and the cell pellet was resuspended in residual media. Inactivation was performed with 0.3% formalin (*v*/*v*) for constant agitation at 4 °C for 48 h. Following, the vaccine formulation was prepared using the oil-based adjuvant Montanide^TM^ ISA 761 VG at a ratio of 30% aqueous suspension to 70% adjuvant, resulting in a final dose volume of 100 µL. The total vaccine volume per group was calculated based on 15 fish per group (5 mL total vaccine per group). The aqueous phase, corresponding to 30% of the formulation, included: phosphate-buffered saline (PBS) for the negative control (C−); cell extract infected with wild-type baculovirus (Bac-WT); coinfection-derived cell extract expressing VLP6n (VLP6n); and coinfection-derived cell extract expressing VLP6c (VLP6c). To prepare the emulsions, 50 mL conical tubes pre-chilled on ice were used. The adjuvant was first added to the tubes. The emulsification process was initiated using an Ultra-Turrax homogenizer at 1000 rpm, with the pre-chilled antigen gradually added, followed by homogenization at 10,000 rpm for 3 min until a stable emulsion was formed. After emulsification, a drop test was performed to assess emulsion quality. The final emulsions were stored at 4 °C until further use.

### 2.12. In Vivo Immunization and Challenge Assays in Atlantic Salmon with Native VLP

The animal trial was outsourced by VESO-Chile company according their animal protocols. The Atlantic salmon, weighing 50 ± 0.5 g, was provided by the Bencmark Chile Spa. They were acclimated at 16 °C under laboratory conditions for 2 weeks before experimental manipulation, then were maintained in aerated water and fed daily with commercial dry feed pellets. During this observation period, the Atlantic salmon did not display any clinical signs. Additionally, fish samples were negative for PRV-1 by qRT-PCR. The Atlantic salmon were divided randomly into three groups (50 fish/group) and injected intraperitoneally for the experiments. Two different formulations using native VLP were tested: VLP6n_1, formulated with antigen normalized to 0.5 μg of σ1 protein, and VLP6n_2, formulated with antigen normalized to 1.5 μg of σ1 protein. A negative group vaccinated with PBS and vehicle was also included. The smoltification process lasted 5 weeks, during which the fish adapted to seawater conditions at 12 °C. At 50 days post-vaccination, fish were challenged with 100 μL with 1 × 10^9^ RNA viral copies of PRV-1 via the intraperitoneal route. The inoculum was obtained from red blood cells with PRV-1 originated from an outbreak of HSMI in Nor-Trøndelag 2012 and passed through fish in VESO Chile. The isolate used was Jno.1911 and the infectious dose was determinate by RT-qPCR using the Genesig Piscine Reovirus kit and a standard RNA curve of known concentration. No mortality was observed in any group during the vaccination or post-challenge periods, and all 50 fish per group were maintained until the 5-week endpoint for blood sampling, randomization was used to allocate animals to control and treatment groups through simple random allocation. Blood tissue samples were collected at 3-, 4-, and 5-week post-challenge. On each sampling whole blood cells were heparinized and placed in RNALater.

### 2.13. PRV-1 RNA Extraction and Titration by RT-qPCR

PRV-1 titers were determined by RT-qPCR. Briefly, total RNA from bloods cells was extracted using the FlavoPrep Tissue Total RNA Purification (Favorgen Pingtung, Taiwan) kit following the manufacturer’s instructions. PRV segment S1 copy number per mg of weighted whole blood cells was determined using the Genesig Piscine Reovirus kit (Primerdesign, Manchester, UK) using a standard curve of known RNA concentration.

### 2.14. In Vivo Immunization Assay in Rainbow Trout with Native VLP and Cmyc VLP

The rainbow trout immunization assay was performed in the experimental unit of Veterquimica SA (Cerrillos, Santiago de Chile). The rainbow trout (*Oncorhynchus mykiss*) weighing 40 g were maintained in freshwater aquariums with the following parameters: 14 °C, 5.8–9 mg/L dissolved oxygen, pH 6.6–6.9, and 3–5% *w*/*v* salinity. Before the immunization assay began, a health check was conducted during the acclimatization period to identify possible pathogens, including IPNV, ISAV, and PRV-1. For this purpose, total RNA was extracted from pooled gill and blood samples of randomly selected fish and analyzed by RT-PCR for IPNV, ISAV, and PRV-1 following SERNAPESCA’s (Chile) diagnostic protocols for viral pathogens. All tests were negative, confirming pathogen-free status before vaccination. A total of 148 fish were used, divided into four groups of 37 individuals each. These four groups were as follows: Group 1: immunized with native VLP (VLPn); Group 2: immunized with VLPcmyc (VLPc); Group 3: immunized with wild-type baculovirus (Bac-WT); and Group 4: negative control immunized with PBS (C-). All fish from each group were anesthetized by benzocaine chloride (0.5 g/10 L of water) immersion and vaccinated intraperitoneally with 100 µL of vaccine formulation containing 1.5 µg of antigen (σ1) corresponding to either native VLP (VLPn) or VLP cmyc (VLPc). The negative control group was injected intraperitoneally with PBS containing an oil-based adjuvant, and another Bac-WT control group was injected with 100 µL of the formulation containing wild-type baculovirus. The immunization was administered as a single dose, and a 10% mortality rate was considered possible. At each sampling point (32, 43, and 64 days post-immunization), 10 individuals per group were randomly selected and euthanized for blood collection and antibody detection, randomization was used to allocate animals to control and treatment groups through simple random allocation This sampling design allowed consistent statistical representation across time points.

### 2.15. Serum Antibody Detection in Vaccinated Trout

Whole blood from each fish was collected and allowed to coagulate at room temperature for 1 h before being centrifuged at 1500× *g* for 5 min to separate the serum. Antibodies in serum samples were detected by ELISA. For this, a 96-well plate was coated with either 100 ng of the cmyc peptide (Thermo Fisher) or 500 ng of the recombinant σ1-His protein. The recombinant σ1-His protein was produced and purified by GenScript Co. (Piscataway, NJ, USA) under standard expression and purification protocols. The protein was expressed in *E. coli* and purified by nickel-affinity chromatography (>90% purity verified by SDS-PAGE). Plates were incubated overnight at 4 °C, washed with PBS, and blocked with 0.5% BSA in PBS for 2 h at room temperature. Following blocking, the plates were washed three times with PBS, and diluted serum samples (1:200) were added for a subsequent 1 h incubation at room temperature. After serum incubation, plates were washed three times with PBS and incubated with 1:500 monoclonal anti-salmon IgM antibody (Ango Co. San Ramon, CA, USA) at room temperature for 30 min. Then, the excess antibody was washed away, and 1:5000 of secondary HRP-conjugated anti-mouse antibody (Thermo Fisher) was added for 30 min at room temperature. Then, plates were washed three times with PBS, and samples were developed with TMB (Invitrogen); the reaction was stopped by adding 100 µL of 1 M hydrochloric acid. Absorbance was determined at 450 nm using a Nanoquant Infinite M2000 Pro plate reader (TECAN, Salzburg, Austria).

### 2.16. Statistical Analysis

All experiments were conducted in triplicate using three biological replicates per group. Statistical differences were analyzed using one-way ANOVA followed by Tukey’s multiple-comparison test (GraphPad Prism 6.0), with *p* < 0.05 considered significant.

## 3. Results

### 3.1. Recombinant Baculovirus Production

**Figure 1:**
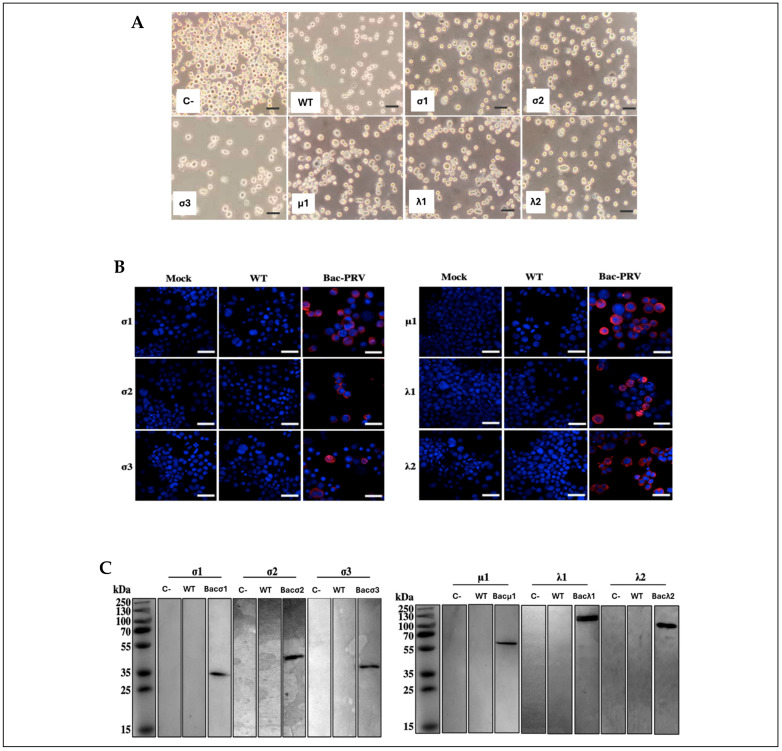
Heterologous PRV-1 protein expression in SF9 cells infected with recombinant baculovirus. (**A**) Optical microscopy of SF9 cells infected with recombinant baculovirus; C-: non-infected cells; WT: wildtype virus; σ1, σ2, σ3, μ1, λ1, λ2: baculovirus-infected cells. (**B**) Representative indirect immunofluorescence pictures of non-infected SF9 cells (mock), cells infected with a wild-type baculovirus (WT), and cells infected with recombinant baculoviruses expressing the PRV structural proteins (Bac-PRV). SF9 cells were infected with the recombinant baculoviruses, and at 3 dpi, protein expression was evaluated by immunofluorescence using specific anti-λ1, anti-λ2, anti-μ1, anti-σ2, anti-σ3, or anti-σ1 antibodies (red). Cell nuclei were stained with DAPI and are shown in blue. Scale bar represents 100 μm. (**C**) PRV internal and external capsid proteins expression in SF9 cells infected with recombinant baculoviruses was assessed by Western blot. C-: cell lysate from non-infected SF9 cells. WT: protein extract from cells infected with a WT baculovirus. Bac-: protein extract from cells infected with recombinant baculoviruses expressing PRV structural proteins.

To produce the recombinant baculoviruses, the SF9 cells were first transfected with recombinant bacmids, then 72 h post-transfection, the SF9 cells showed enlarged nucleus and high cell size. At 5 days post-transfection, all samples exhibited CPE, noted as enlarged cells and nucleus. Secondly, the transfection supernatant was used to infect SF9 cells (Figure 1A). After 3 days post-infection, the recombinant protein expression was assessed by IFA in SF9-infected cells. This analysis (Figure 1B) revealed no PRV-1 protein detected in both the mock-infected cells and cells infected with a wild-type (WT) baculovirus control. But PRV-1 structural proteins were detected in infected cells with recombinant baculoviruses. After confirming recombinant baculoviruses rescue and their ability to express the desired proteins, it was further verified by Western blot (Figure 1C). As expected, no detection was observed in the cellular lysate of mock-infected cells and cells infected with a WT baculovirus. On the other hand, bac-λ1-, bac-λ2-, bac-μ1-, bac-σ2-, bac-σ3-, and bac-σ1-infected cells, showed bands at 141.5 kDa, 143.7 kDa, 74.2 kDa, 45.9 kDa, 37.0 kDa, and 36.4 kDa, respectively. These results confirm that SF9 cells infected with the recombinant baculoviruses express the PRV-1 structural proteins as expected, with molecular sizes concordant with previous literature [6].

### 3.2. Analysis of σ1-Cmyc Recombinant Protein Expression

**Figure 2:**
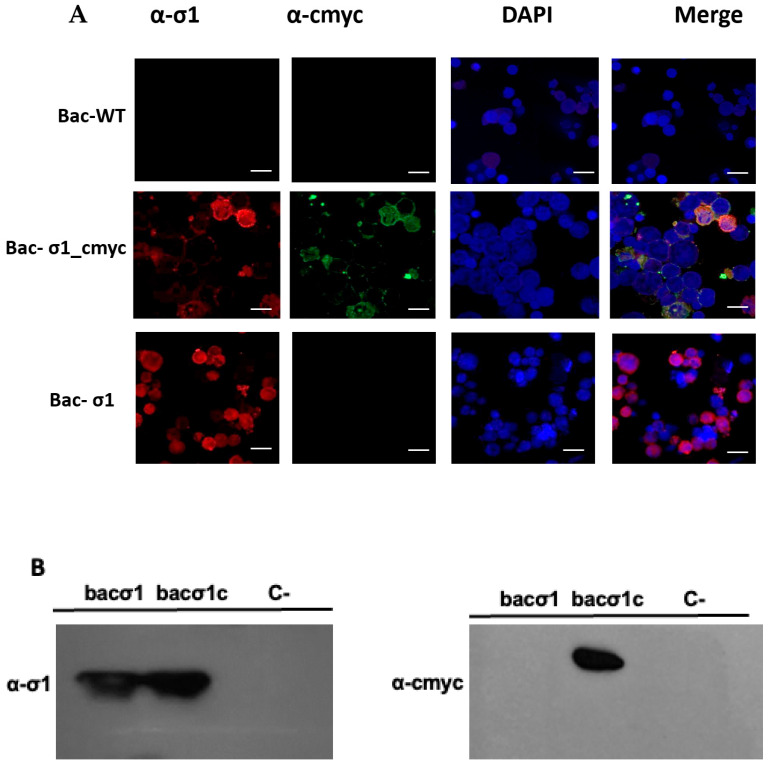
Detection of σ1-cmyc Recombinant Protein. (**A**) Indirect Immunofluorescence Assay (IFA) for the co-detection of the σ1 protein and the cmyc epitope. SF9 cell monolayers were infected with the indicated recombinant baculoviruses. At 3 dpi, infected cells were fixed, stained, and incubated with the indicated antibodies. Secondary antibodies for σ1 and the cmyc epitope were donkey anti-rabbit Alexa Fluor 594 (red) and donkey anti-mouse Alexa Fluor 488 (green), respectively, and the nucleus was counterstained with DAPI (blue). WT corresponds to cells infected with a wild-type baculovirus. Scale bar represents 100 μm. (**B**) Detection of recombinant σ1 and σ1cmyc proteins by Western blot. The σ1 and cmyc epitopes were detected with anti-σ1 and anti-cmyc antibodies; C- indicates non-infected cells.

Expression of the σ1-cmyc protein was evaluated by immunofluorescence in insect cells. In Figure 2A, both σ1 and cmyc were detected using specific antibodies to observe a colocalization (Figure 2A). An overlap between the two proteins was observed, therefore indicating the colocalization of σ1 and the cmyc epitope. No signal was observed in cells infected with a wild-type baculovirus (Figure 2 Line Bac-WT). Then, the cmyc epitope and σ1 protein were detected by western blot (Figure 2B) using anti-cmyc and anti-σ1 antibodies. On the σ1-cmyc protein, positive immunodetection was performed using both anti-σ1 and anti-cmyc antibodies, indicating that the cmyc linear peptide is part of the molecular structure of the σ1 protein.

### 3.3. Co-Expression of Recombinant Proteins and VLP Visualization

**Figure 3:**
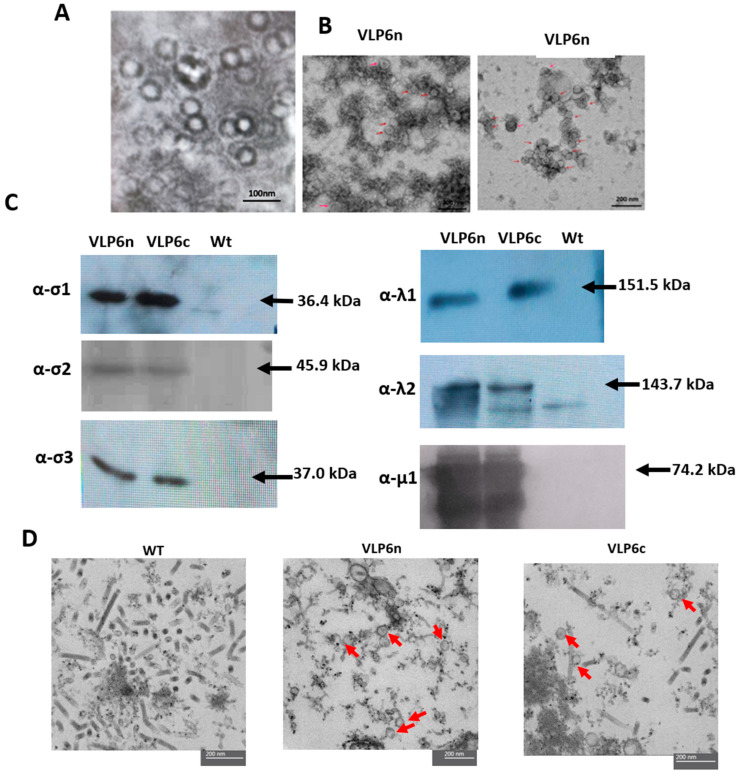
Characterization of purified VLP by TEM and co-infection cell extract characterization by immunodetection and TEM. (**A**) Electron micrographs showing PRV-1-like (empty) particles purified from co-infected insect cells using sucrose gradient, scale bar 100 nm (~60–80 nm). (**B**) Electron micrographs showing PRV-1-like (red arrows) particles purified from co-infected insect cells using CsCl gradient, scale bar 200 nm. VLP6n and VLP6c correspond to isolated particles from cells co-infected with recombinant proteins σ1, σ2, σ3, μ1, λ1, and λ2 to form native σ1 viral particles, and cells co-infected with recombinant proteins σ1cmyc, σ2, σ3, μ1, λ1, and λ2 to form modified empty viral particles with σ1cmyc, respectively. (**C**) Immunodetection of recombinant proteins σ1, σ2, σ3, μ1, λ1, and λ2 in co-infected cell extracts. Detection of proteins σ1, σ2, σ3, λ1, and λ2 was performed using specific antibodies: anti-σ1, anti-σ2, anti-σ3, anti-λ1, and anti-λ2, respectively. VLP6n and VLP6c correspond to cells co-infected with recombinant proteins σ1, σ2, σ3, μ1, λ1, and λ2 to form native σ1 viral particles, and cells co-infected with recombinant proteins σ1cmyc, σ2, σ3, μ1, λ1, and λ2 to form modified empty viral particles with σ1cmyc, respectively. (**D**) Electron micrographs of ultrathin sections of insect cells infected with baculoviruses expressing recombinant proteins σ1 or σ1cmyc, σ2, σ3, λ1, λ2, and μ1. VLP6n and VLP6c correspond to co-infection cell extract samples with bac-λ1, bac-λ2, bac-μ1, bac-σ2, bac-σ3, bac-σ1 and bac-λ1, bac-λ2, bac-μ1, bac-σ2, bac-σ3, bac-σ1cmyc, respectively. The WT control corresponds to wild-type baculovirus infection. Scale bar = 200 nm. Red arrows indicate PRV-1-like particles.

Co-infection assays were performed to analyze the VLP production. High Five cells were co-infected with baculoviruses expressing the σ1, σ1-cmyc, σ2, σ3, μ1, λ1, and λ2 proteins, and at 5 days post-infection, co-expression of these proteins was determined by western blot (Figure 3C). This assay showed bands of the expected size for each protein, confirming the simultaneous production of the σ1, σ1-cmyc, σ2, σ3, μ1, λ1, and λ2 proteins in the same cell extract. Once the simultaneous production of the recombinant proteins was confirmed, the next goal was to determine their ability to self-assemble into PRV-1-like particles. To determine VLP’s formation, the native VLP were purified by ultracentrifugation and collected at the interface between 30 and 50% sucrose concentrations. In the same way, after CsCl gradient, the viral particles were collected from the whitish band, as reported by Wessel et al. [8]. The results showed particles that resembled VLP by retaining a diameter between 60–80 nm (Figure 3A) Additionally, VLP visualizations was better using CsCl gradient purification (Figure 3B, red arrows). Then, in order to confirm these results, the ultrathin section results by TEM images (Figure 3D) revealed that VLP were formed when cells were co-infected with either the bac-λ1, bac-λ2, bac-μ1, bac-σ2, bac-σ3, bac-σ1 (VLP6n) or the bac-λ1, bac-λ2, bac-μ1, bac-σ2, bac-σ3, bac-σ1cmyc (VLP6c) combination. Red arrows indicate the presence of VLP, ranging from 60–80 nm in size and with an icosahedral geometry, similar to what has been reported for PRV-1 [5]. These structures were not observed in infected cells with wild-type baculovirus (Figure 3D, WT). In addition to observing these VLP assemblies, dense, elongated, and circular structures were abundantly visible, with sizes ranging from 100 to 250 nm and 20 nm, respectively (Figure 3D, red arrows in VLP6n and VLP6c; scale bar indicates 100–250 nm). This size is consistent with the expected size of baculovirus budding particles, which are cylindrical structures measuring 260 nm in length and 20 nm in diameter. Considering the observation of viral-like structures with size and morphology similar to PRV-1 (with a 70 nm diameter), these results indicate that co-expressed PRV-1 structural proteins can form PRV-1-like particles (60–80 nm) with morphology consistent with previous reports. However, definitive proof of complete assembly was not obtained.

### 3.4. PRV-1 Challenge and Viral Load Analysis in Vaccinated VLP6n Atlantic Salmon

**Figure 4:**
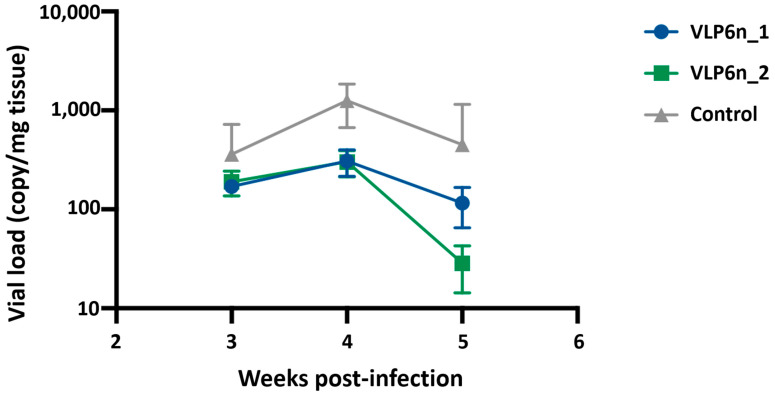
Infection kinetics in Atlantic salmon vaccinated with the VLP6n and adjuvant formulation. Quantification of viral titers (copy/mg blood tissue) from the blood samples at 3, 4, and 5 weeks post-infection in vaccinated fish challenged with PRV-1. A control group vaccinated with PBS is included.

After confirming co-expression of proteins in the cell extract and observing PRV-1-like viral particles by electron microscopy, in vivo assays were conducted using vaccine formulations (Figure 4). The first vaccine prototype, formulated with a cell extract containing native PRV proteins (VLP6n), was evaluated for its protective efficacy against PRV-1 infection. Atlantic salmon were vaccinated using two formulations: VLP6n_1, which consisted of 1.5 µg of σ1 antigen, and VLP6n_2, which had 0.5 µg of σ1 antigen. After 50 days post-immunization, salmon were challenged with PRV-1. Following the challenge, whole blood cells samples were collected, and viral loads were determined in immunized fish and mock-vaccinated fish. These results demonstrated that at 5 weeks post-infection, viral loads in vaccinated fish were significantly lower compared to the mock-vaccinated group, with the lowest value observed in fish immunized with VLP6n_2, which reduced the PRV copy number per milligram of tissue by approximately 16 times compared to the non-vaccinated control (Figure 4). Therefore, formulations tested in this assay exhibited protection against PRV in a concentration-dependent manner, with the formulation containing the highest protein concentration being the most effective.

### 3.5. Immunization of Rainbow Trout Using Native-VLP and Cmyc-VLP

**Figure 5:**
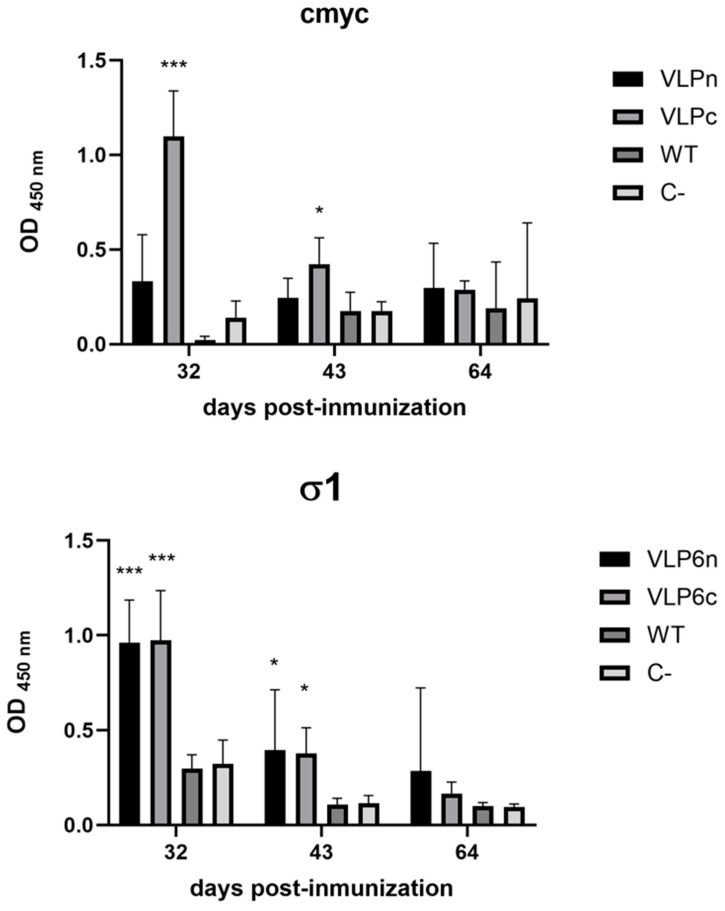
Comparison of IgM antibody levels generated against the σ1 proteins and the cmyc peptide in the vaccinated groups (VLP6n, VLP6c, Bac-WT, and C-) through indirect ELISA. The σ1 graph shows IgM antibody levels against σ1 in fish sampled at 34, 43, and 64 days post-immunization. Microtiter plates were coated with 500 ng of recombinant σ1-His protein (σ1 graph) or 100 ng of cmyc peptide (cmyc graph) and incubated with a 1:100 dilution of fish serum from each group. A 1:1000 dilution of monoclonal anti-salmon IgM antibody was then used, followed by incubation with a secondary anti-mouse antibody. The analyzed groups were VLP6n, VLP6c, Bac-WT, and C-, corresponding to the formulations with co-infection cell extracts bac-λ1, bac-λ2, bac-μ1, bac-σ2, bac-σ3, and bac-σ1; bac-λ1, bac-λ2, bac-μ1, bac-σ2, bac-σ3, and bac-σ1cmyc; wild-type baculovirus; and PBS. Statistical analysis corresponds to ANOVA. * *p* < 0.05; *** *p* < 0005.

To determine the DIVA attribute of VLPs, vaccine rainbow trout were injected with VLP6n and VLP6c cellular extracts. Thus, to evaluate the capacity of co-infection cell extracts to induce an immune response against PRV-1 and the cmyc epitope, an immunization trial was performed in rainbow trout (*Oncorhynchus mykiss*), selected due to fish availability at the time of the experiment and their established use in immunological studies for salmonids, supported by previous research [19,20]. The trial utilized an injectable emulsion formulation containing, in addition to five other PRV recombinant proteins, 1.5 µg of either σ1 or σ1cmyc. In this case, the VLP6n and VLP6c formulations were used. Additionally, two control groups were included in the trial: Group Bac-WT, which was immunized with wild-type baculovirus cell extract, and Group C-, which was inoculated with PBS. At 34, 43, and 64 days post-immunization, serum was collected, and the ability to induce an immune response against cmyc and σ1 was evaluated by ELISA. For this purpose, sera from each group were used, and ELISA plates were coated with either recombinant protein σ1 or the cmyc peptide. ELISA results (Figure 5) showed that at 34 days post-immunization, antibody production against σ1 differed between the VLP6n and VLP6c groups compared with the Bac-WT and C groups. An absorbance of 0.9, 1.7, 0.4, and 0.3 was detected in the VLP6n, VLP6c, Bac-WT, and C-groups, respectively, suggesting that both formulations (VLP6n and VLP6c) induced the production of anti-σ1 antibodies in the rainbow trout tested. This difference was smaller until 43 days post-immunization. Subsequently, when the anti-cmyc response was assessed, significant differences were observed between the VLP6c and VLP6n groups at 32 days post-immunization, with absorbance values of 0.2 and 1.1, respectively. Significant differences were also observed between VLP6c compared to Bac-WT and C-, with absorbance values of 1.1, 0.1, and 0.3 for the VLP6c, Bac-WT, and C- groups, respectively. These results demonstrated that cmyc epitope was recognized by the host immune system, but the specific antibody response is observed up to 32 days post-immunization.

## 4. Discussion

The *piscine orthoreovirus* has been considered an emergent pathogen, threatening salmon production. It has become the most prevalent virus in Norway, reaching higher prevalence in farms [21]. In the aquaculture industry, vaccination is a widely used tool to control viral infections. Currently, VLP-based vaccines for fish viral diseases, including infectious pancreatic necrosis virus (IPNv), salmon alpha virus (SAV), and nervous necrosis virus (NNV), have been developed and tested [22]. Nonetheless, different vaccine strategies have been used against PRV, including an inactivated vaccine [8] and a DNA vaccine [10]. To our knowledge, this is the first report describing a PRV-1 VLP-based vaccine.

In our study, native-VLP and cmyc-VLP PRV-1 vaccines were made using a baculovirus-based expression system. The σ1-cmyc chimeric protein was generated by inserting the cmyc oncogene into the Sigma 1 ORF and cloned into the pFastBac-σ1 plasmid. This plasmid was used to produce a recombinant baculovirus expressing the aforementioned chimeric protein. Western blot immunodetection analysis of bac-σ1cmyc using anti-σ1 and cmyc antibodies showed bands at approximately 35 kDa (Figure 2B), which demonstrated the presence of the σ1 protein and the cmyc epitope in the cell extract infected with bac-σ1cmyc. Additionally, the Indirect Immunofluorescence Assay (IFA) analyses of bac-σ1cmyc revealed an overlap of σ1 and cmyc fluorescent signals (Figure 2A), further confirming the successful incorporation of the cmyc epitope into the σ1 protein. The λ1, λ2, μ1, σ2, σ3, σ1, and σ1cmyc proteins spontaneously self-assembled into VLP bearing either a native σ1 (VLP6n) or σ1-cmyc VLP (VLP6c). Both VLPs were observed in ultrathin sections from co-infection extracts. Although particles resembling PRV-1 were observed, further studies using antibody-coupled TEM or proteomics are required to confirm that all structural proteins are incorporated. Therefore, these assemblies are referred to here as PRV-1-like particles.VLP6n and VLP6c were similar in size and morphology compared to the wild-type virus, as previously described [8]. The baculovirus–insect cell expression system has previously been used to produce recombinant proteins for the formation of virus-like particles (VLPs) from other fish viruses, such as grass carp reovirus [13,23] and salmon alpha virus [24,25], demonstrating the system’s efficiency for VLP generation. Our results are consistent with previous VLP-based vaccines developed for aquaculture viruses, such as GCRV-II in grass carp [13] and IPNV in salmonids [15], which demonstrated strong antibody induction and protection without using live virus. These findings further support the suitability of the baculovirus-insect cell system for scalable and safe VLP vaccine production.

It is noteworthy that the expression temperature can influence protein conformation and particle assembly; for instance, lower culture temperatures (e.g., 20–25 °C) have been reported to enhance proper protein folding and improve VLP assembly in some systems [26]. Although we cultured the cells at 28 °C in this study and did not specifically investigate temperature effects, future studies could explore expression at reduced temperatures to potentially increase the fidelity of VLP assembly.

PRV infection is characterized by an increase in viral titers in blood and plasma throughout infection, about 6 weeks [2,6], resembling our observations in the non-vaccinated control group. The vaccinated group exhibited significantly lower blood titers after challenge, highlighting the substantial impact of vaccination on protection. Furthermore, a dose-dependent response was observed, with the most concentrated formulation (VLP6n_2) proving to be the most effective vaccine. Virus quantification in the primary site of infection or other target organs can account for protection. During infection, a confirmed increase in viral load in the blood has been observed in the blood [27]. The trial duration (5 weeks) was designed to capture the acute infection phase, as PRV-1 peaks in tissues during this period [1,5]. Future trials will extend monitoring to evaluate long-term cardiac pathology and correlate with antibody responses. As observed in previous studies [8,10], vaccine protectiveness can be correlate with lower viral RNA levels in blood, reflecting related to a low number of viral particles affected by vaccination. The observation of lower viral load levels in challenged salmon in the high-dose vaccine group could suggest a decreased likelihood of viral shedding. This reduction in viral excretion is crucial for containing the spread of *Piscine Orthoreovirus* (PRV-1) infection, a highly contagious pathogen that can reach 100% prevalence in infected farms.

Vaccine immunogenicity was evaluated by looking at the production of serum IgM-specific antibodies targeting the cmyc epitope and the σ1 protein. IgM antibodies constitute a significant immunoglobulin serotype present in salmonids and can act as an immune response marker [28]. Given the knowledge of functional activation of IgM^+^ b cells in rainbow trout and its suitability for antibody detection [29], this species was an appropriate model to evaluated the immunogenicity associated with the DIVA attribute.

It is important to note that PRV susceptibility differs among salmonid species. Atlantic salmon are primarily affected by PRV-1, which causes HSMI, whereas rainbow trout are mainly susceptible to PRV-3, associated with HSMI-like disease and anemia [30,31]. Despite these subtype differences, both species share conserved immune mechanisms [5], making rainbow trout a relevant comparative model for evaluating humoral responses under controlled laboratory conditions.

Previous studies have shown that PRV genotype 3 (PRV-3), which predominates in rainbow trout and coho salmon in Chile, can induce partial cross-protection against PRV-1, the genotype responsible for HSMI in Atlantic salmon in Norway [5,31]. This cross-reactivity likely arises from conserved epitopes within the σ1 and μ1 structural proteins. Based on these findings, our PRV-1-derived VLP vaccine may serve as a foundational model for the development of multi-genotype vaccines, particularly in regions where PRV-3 and PRV-1 co-circulate. Incorporating this epidemiological context highlights the broader applicability of VLP-based vaccination strategies across diverse salmonid species.

This study focused on early-stage immunogenicity and did not assess the duration of immunity or the potential requirement for booster vaccination. Furthermore, the scalability of VLP production and the inclusion of antigens from additional PRV genotypes (such as PRV-3) represent key priorities for future optimization aimed at achieving broad-spectrum protection in salmonids

The antibody response data obtained in trout complement the protection results observed in Atlantic salmon challenged with PRV-1. Following immunization with either the native-VLP or the cmyc-VLP vaccine, anti-σ1 antibody levels increased significantly at 34 days post-vaccination compared with the control groups (Bac-WT and C−). However, antibody titers declined at both 43 and 64 days post-immunization, suggesting that a prime–boost regimen may be required to sustain IgM levels over time.

This decline in IgM titers represents a typical transient primary antibody response in salmonids, as previously reported in fish vaccination studies [32]. It highlights the potential need for a booster dose to prolong humoral immunity.

Additionally, we observed that the σ1-specific antibodies induced by the cmyc-VLP were higher compared to those induced by the native-VLP. Enhanced antibody production could suggest that the cmyc change the σ1 protein’s antigenicity. To date, two studies have reported novel vaccine candidates against PRV-1 [8,10]; but this studies did not evaluated IgM production. Nevertheless, our findings demonstrate that both the native-VLP and cmyc-VLP IgM against σ1 in vaccinated trout.

To explore the suitability of our vaccines for DIVA strategies, we evaluated anti-cmyc IgM antibodies in serum samples from animals vaccinated with the VLP-cmyc vaccine. Significant differences were observed between the cmyc-VLP and native-VLP vaccine groups, with the highest anti-cmyc IgM levels observed in the cmyc-VLP group at 32 days of immunization. In a previous study, the cmyc tag was utilized to produce cmyc subviral particles (cmyc-SVP) against Infectious Pancreatic Necrosis Virus (IPNV) [33]. Other data showed that VP2 tolerated the insertion of cmyc without affecting SVPs’ morphology or their antigenicity. This contrasts with previous reports in which no significant differences in antibody production between labelled and native antigens had been observed [34,35,36]. Additionally, since the σ1 protein was proved here to be a flexible antigen, supporting the introduction of small foreign epitopes, it makes it a promising scaffold candidate to carry new epitopes from other pathogens. This would help with the development of multivalent or multi-epitope vaccines.

In summary, the present study demonstrated that a native-VLP vaccine significantly reduced PRV-1 viral load in salmon. Additionally, the introduction of a foreign epitope (cmyc) elicited a specific humoral anti-cmyc response, which makes this platform suitable for DIVA strategies. This vaccine platform could serve as a potential candidate with promising development prospects for controlling PRV-1 prevalence in salmon-producing countries.

This study was conducted under controlled laboratory conditions; therefore, future field trials are essential to evaluate vaccine performance under farming conditions. Furthermore, the scalability and process optimization of PRV-1 VLP must be addressed to assess their feasibility for large-scale manufacturing. These steps will be critical for translating laboratory findings into a commercially viable vaccine solution for salmonid aquaculture.

## 5. Patents

This work results in a patent request applied to INAPI-CHILE with number #227-2024 and to PCT as PCT/CL2025/050007. Number of publication WO/2025/156063.

## Data Availability

The original contributions presented in this study are included in the article. Further inquiries can be directed to the corresponding author.
